# Supplementary material for: Splice-Junction-Based Mapping of Alternative Isoforms in the Human Proteome
Source: Cell Rep. Author manuscript; Available in PMC 2020 Jan 15. (PMC6961840; doi:10.1016/j.celrep.2019.11.026)
Supplement: 2 [file NIHMS1546469-supplement-2.zip › DF1/PXD010154/Ovary/Ovary_20_MLX_LDPWVKASPVGAHAGEGR.pdf]

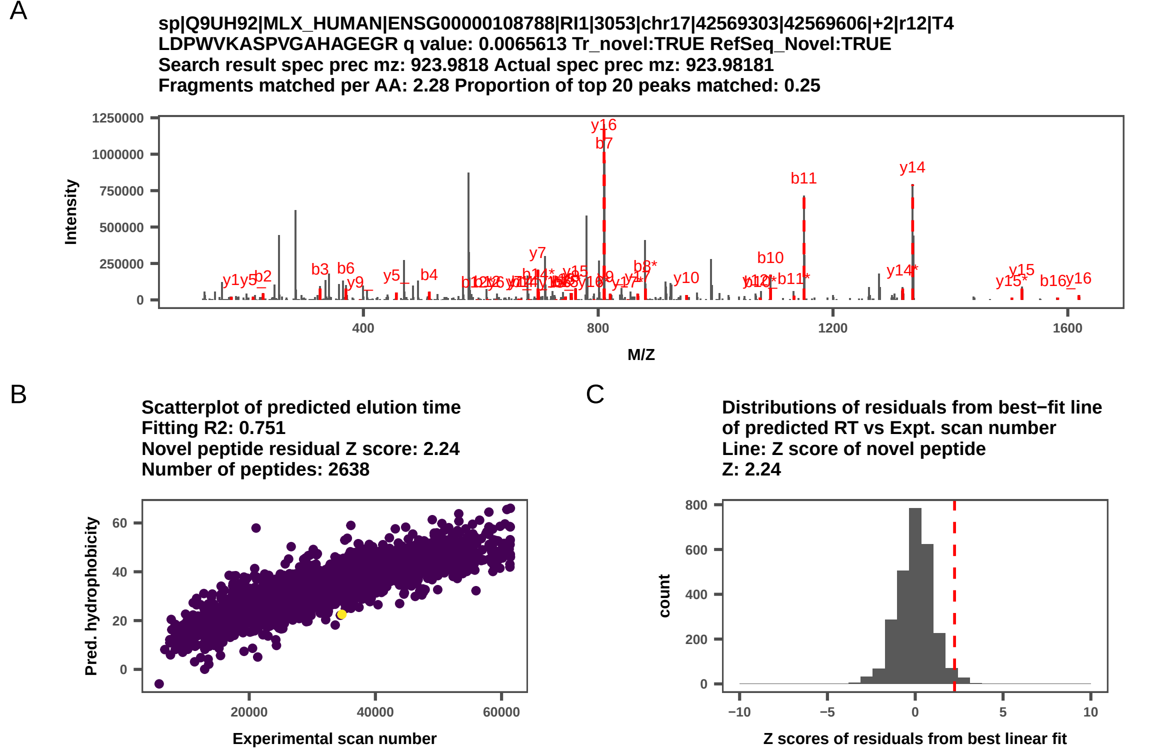
A
sp|Q9UH92|MLX_HUMAN|ENSG00000108788|RI1|3053|chr17|42569303|42569606|+2|r12|T4
LDPWVKASPVGAHAGEGR q value: 0.0065613 Tr_novel:TRUE RefSeq_Novel:TRUE
Search result spec prec mz: 923.9818 Actual spec prec mz: 923.98181
Fragments matched per AA: 2.28 Proportion of top 20 peaks matched: 0.25
Intensity
1250000
1000000
750000
500000
250000
0
M/Z
400
800
1200
1600
y16
b7
b11
y14
b10
y7
b3
b6
b4
y10
y14*
y15
y15*
b16
y16
b8*
b11*
B
Scatterplot of predicted elution time
Fitting R2: 0.751
Novel peptide residual Z score: 2.24
Number of peptides: 2638
Pred. hydrophobicity
60
40
20
0
20000
40000
60000
Experimental scan number
C
Distributions of residuals from best−fit line
of predicted RT vs Expt. scan number
Line: Z score of novel peptide
Z: 2.24
count
800
600
400
200
0
−10
−5
0
5
10
Z scores of residuals from best linear fit
